# A homozygous *PIWIL2* frameshift variant affects the formation and maintenance of human-induced pluripotent stem cell-derived spermatogonial stem cells and causes Sertoli cell-only syndrome

**DOI:** 10.1186/s13287-022-03175-6

**Published:** 2022-09-24

**Authors:** Xiaotong Wang, Zili Li, Mengyuan Qu, Chengliang Xiong, Honggang Li

**Affiliations:** 1 Institute of Reproductive Health/Center of Reproductive Medicine, Tongji Medical College, Huazhong University of Science and Technology, Wuhan, 430030 China; 2 Wuhan Tongji Reproductive Medicine Hospital, Wuhan, 430013 China; 3 Hubei Engineering Research Center for Preparation, Application and Preservation of Human Stem Cells, Wuhan, 430013 China; 4 The Third Affiliated Hospital of Zhengzhou University, Zhengzhou, 450052 China

**Keywords:** Male infertility, Sertoli cell-only syndrome, *PIWIL2*, Induced pluripotent stem cell, Spermatogonial stem cell

## Abstract

**Background:**

The most serious condition of male infertility is complete Sertoli cell-only syndrome (SCOS), which refers to the lack of all spermatogenic cells in the testes. The genetic cause of SCOS remains to be explored. We aimed to investigate the genetic cause of SCOS and assess the effects of the identified causative variant on human male germ cells.

**Methods:**

Whole-exome sequencing was performed to identify potentially pathogenic variants in a man with complete SCOS, and Sanger sequencing was performed to verify the causative variant in this man and his father and brother. The pathogenic mechanisms of the causative variant were investigated by in vitro differentiation of human-induced pluripotent stem cells (hiPSCs) into germ cell-like cells.

**Results:**

The homozygous loss-of-function (LoF) variant p.His244ArgfsTer31 (c.731_732delAT) in *PIWIL2* was identified as the causative variant in the man with complete SCOS, and the same variant in heterozygosis was confirmed in his father and brother. This variant resulted in a truncated PIWIL2 protein lacking all functional domains, and no PIWIL2 expression was detected in the patient’s testes. The patient and *PIWIL2*^−/−^ hiPSCs could be differentiated into primordial germ cell-like cells and spermatogonial stem cell-like cells (SSCLCs) in vitro, but the formation and maintenance of SSCLCs were severely impaired. RNA-seq analyses suggested the inactivation of the Wnt signaling pathway in the process of SSCLC induction in the *PIWIL2*^−/−^ group, which was validated in the patient group by RT-qPCR. The Wnt signaling pathway inhibitor hindered the formation and maintenance of SSCLCs during the differentiation of normal hiPSCs.

**Conclusions:**

Our study revealed the pivotal role of PIWIL2 in the formation and maintenance of human spermatogonial stem cells. We provided clinical and functional evidence that the LoF variant in *PIWIL2* is a genetic cause of SCOS, which supported the potential role of *PIWIL2* in genetic diagnosis. Furthermore, our results highlighted the applicability of in vitro differentiation models to function validation experiments.

**Supplementary Information:**

The online version contains supplementary material available at 10.1186/s13287-022-03175-6.

## Background

Male infertility affects approximately 7% of the male population [1], and azoospermia cases represent 10–20% of male infertility cases [2]. Non-obstructive azoospermia (NOA) is a spectrum of spermatogenic quantitative defects, which accounts for the majority of azoospermia cases (> 70%) and affects 1% of the adult male population [2, 3]. NOA includes three patterns of testicular histology, hypospermatogenesis, maturation arrest, and Sertoli cell-only syndrome (SCOS). SCOS, which affects 26.3%-57.8% of men with azoospermia [4], is characterized by the lack of spermatogenic cells but only Sertoli cells remaining in seminiferous tubules. Complete SCOS is the most serious condition wherein all observed seminiferous tubules of testicular biopsy only contain Sertoli cells; in contrast, in incomplete SCOS, some spermatogenic areas remain in the testes. Men with azoospermia have the highest risk of being affected by genetic anomalies [1]. The etiology of SCOS involves genetic factors. The already known genetic causes that have been applied in the clinical etiologic diagnosis of SCOS are karyotype abnormalities and microdeletions of the azoospermic factor on the Y chromosome. Unfortunately, a large proportion of SCOS cases remain unexplained [5], and the etiology and pathogenesis of SCOS have not yet been fully elucidated.

To gain insights into the etiology and pathogenesis of SCOS, we referred to mouse models with Sertoli cell-only (SCO) phenotype; for instance, *Prdm14*^−/−^, *Tcfap2c *^*flox/flox*^*:Sox2-cre*, *Fancb*^−/−^, *Id4*^−/−^, *Zfp145*^−/−^, and *Wt1*^*−/flox*^*; Cre-ER™* mice have the testicular phenotype similar to that of human SCOS [6–11]. According to these mouse models, abnormalities in primordial germ cells (PGCs), spermatogonial stem cells (SSCs), or Sertoli cells can lead to the absence of spermatogenic cells after birth, resulting in an SCO phenotype. In recent years, whole-exome sequencing (WES) has been performed in men with SCOS from consanguineous and non-consanguineous families, revealing several pathogenic or potentially pathogenic variants in genes, such as *FANCA*, *FANCM*, *TEX15*, *KLHL10*, *DMRT1*, *USP26*, *NANOS2*, *TEX14*, and *WNK3* [3, 12–15]. Among these genes, *FANCM*, *DMRT1*, and *NANOS2* have corresponding gene knockout mouse models that exhibit a phenotype similar to human SCOS [14, 16, 17]. As at least 2,000 genes are involved in spermatogenesis [1] and various mouse models manifest SCO phenotype, new genes implicated in human SCOS are expected to be identified. In addition, germ cell development is an important issue for fertility and inheritance. Hence, identifying genes that can lead to SCOS could be an approach to identify candidate genes involved in human germ cell development.

Because the mouse and human reproductive systems are not identical and genes may have different functions or transmit disease through different modes of inheritance, caution is urged to draw conclusions on gene function and inheritance mode based on mouse models only [18]. Human-induced pluripotent stem cell (hiPSC)-derived somatic cells have been proven to be a new model to study genetic disorders [19–22]. Differentiation of hiPSCs into human male germ cell-like cells could provide a better in vitro disease model to study male infertility [23]. This approach bypasses the ethical and technical problems of using human primary germ cells for functional experiments, and also generates large numbers of primordial germ cell-like cells (PGCLCs) and spermatogonial stem cell-like cells (SSCLCs) in a short time [24–28]. Furthermore, this approach could be an in vitro model to study genotype–phenotype correlations because hiPSCs and differentiated cells retain the patient’s genetic background.

In this study, we applied WES in a man with complete SCOS and identified a homozygous frameshift deletion variant in *PIWIL2* as the genetic cause of SCOS. *PIWIL2* is the ortholog of murine *Mili* and encodes a protein that represses the activity of transposons via piRNA pathway. Mutations of other PIWI family members in male infertility have attracted much attention [29, 30]. Recent studies have also suggested *PIWIL2* as the candidate gene of male infertility in men [31], although without functional validation. MILI and PIWIL2 are restricted to germ cells in testis both in human and in mouse [32–34]. Deletion of *Mili* in mouse causes meiosis arrest or incomplete SCO phenotype without any loss of Sertoli cells or interstitial cells [32, 33], which is not identical to the complete SCOS testicular histology of the patient included in our study. Furthermore, the expression of PIWIL2 in germ cells is different between mice and humans [34]. We suspected that PIWIL2 might have different roles in regulating human germ cell development and spermatogenesis. Using in vitro differentiation models, we found evidence that the identified variant in *PIWIL2* affected the formation and maintenance of SSCLCs, along with dysregulation of the Wnt signaling pathway. These results supported that the loss-of-function (LoF) variant in *PIWIL2* leads to human SCOS.

## Methods

### Participants

**Fig. 1:**
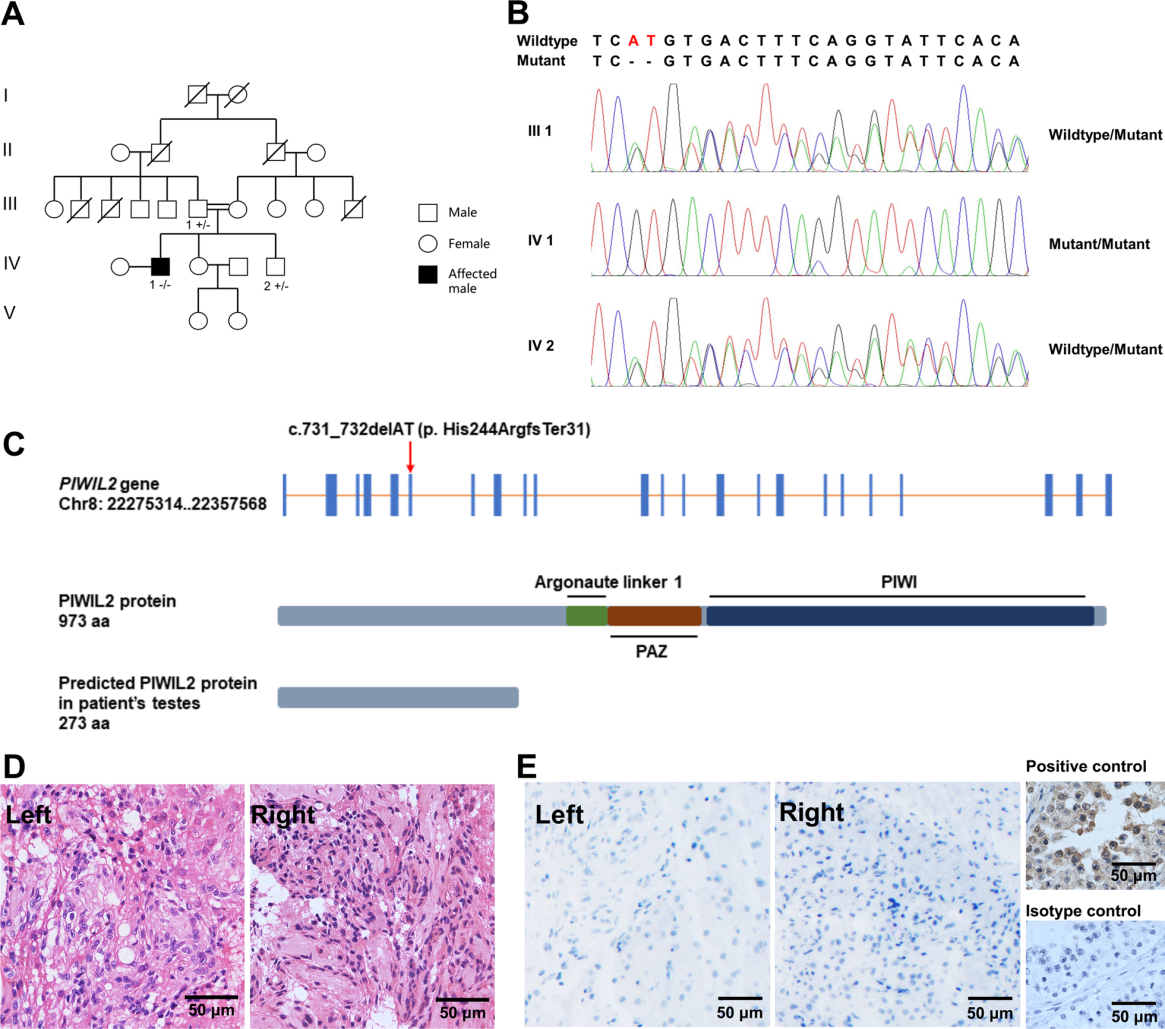
Identification of a homozygous frameshift deletion variant in *PIWIL2* gene in a man with complete SCOS. **A** The patient (IV 1) came from a consanguineous family where his father (III 1) and mother were first-cousins, and his siblings were fertile. **B** Sanger sequencing confirmed the homozygous c.731_732delAT variant in *PIWIL2* in the patient and the same variant in heterozygosis in his father and brother (IV 2). **C** The homozygous c.731_732delAT variant located in exon 6 of *PIWIL2*. This variant introduced a premature stop codon that resulted in a truncated PIWIL2 protein (273 aa), which lacked functional domains of PIWIL2 protein. **D** H&E-stained testicular sections of the patient showed no spermatogenic cells existed in seminiferous tubules and only Sertoli cells arranged on the membrane in all observed seminiferous tubules. **E** Immunohistochemical detection of PIWIL2 with an antibody whose antigen was the middle part of PIWIL2 in testicular sections of the patient and man with normal spermatogenesis. No positive signal was observed in testicular tissue of the patient (Left and Right). PIWIL2 is expressed in spermatogonium, spermatocytes, and spermatids in testicular tissue of man with normal spermatogenesis (Positive control)

We retrospectively reviewed all patients with NOA who had visited our institute between January 13, 2014, and October 8, 2015, to choose patients with complete SCOS according to the following criteria: i) the complete loss of spermatogenic cells in all observed seminiferous tubules in a testicular biopsy and ii) negative seminal *DDX4* mRNA [5]. Patients with known genetic causes, including abnormal karyotype or AZF microdeletions, and those with no results of karyotype or AZF microdeletions tests were excluded. The patient included in the present study was one of the patients with complete SCOS who agreed to provide genomic DNA (gDNA) for WES. He (Fig. 1A, IV1) came from a consanguineous family in which his parents were first-cousins. He had no general health problems. The clinical parameters are listed in Table 1. He and his wife had a child through intrauterine artificial insemination by a donor, suggesting that his wife was fertile. His relatives claimed that they did not suffer from infertility. His younger brother had normal semen analysis results. His sister has two daughters. His father (Fig. 1A, III1) and younger brother (Fig. 1A, IV2) agreed to participate in our study to provide their gDNA. His mother had been disconnected from his family for years, and thus was not included in this study.

**Table 1:** Clinical parameters of the man with complete SCOS included in this study

Parameters	Reference values	Detection values
Age (years)		42
Testis volume: left (mL)	≥ 12	5
Testis volume: right (mL)	≥ 12	5
Testis biopsy		SCOS
Karyotype		46, XY
Microdeletions of Y chromosome		Not detected
*Hormone analysis*		
Total testosterone (nM/L)	10.41–19.78	11.93
Free testosterone (nM/L)	0.11–0.66	0.27
FSH (IU/L)	1.27–19.26	37.72
LH (IU/L)	1.24–8.62	12.56
SHBG (nM/L)	13.2–89.5	25.7
*Semen analysis*		
Semen volume (mL)	≥ 1.5	5.4
Total sperm count (10^6^/ejaculate)	≥ 39	0
Seminal neutral alpha-glucosidase (IU/L)	35.1–87.7	74.55
Seminal fructose (g/L)	2.95–3.35	42.24
Seminal elastase (ng/mL)	< 290	1920.5
Seminal *DDX4* mRNA		Not detected

All of the individuals included in this study signed informed consent.

### Whole-exome sequencing and data analysis

Total gDNA was isolated from peripheral blood using QIAamp DNA Blood Mini Kit (Qiagen, Germany) and passed integrity assessment. After being fragmented with Covaris-focused ultrasonication, DNA was used to prepare a sequencing library using SureSelect Human All Exon v6 Kit (Agilent, USA). Sequencing was performed on an Illumina HiSeq 2500 platform. We obtained 14.2 G of clean data. The percentage of the base above Q30 was 88.1%. The mean sequencing average depth was 123.8 and 20X sequence coverage was 94.3%. The sequencing reads were aligned to the human genome (GRCh37/hg19) using Burrows-Wheeler Aligner before removing adaptors, PCR duplicates, and low-quality reads. Single nucleotide substitutions and indel variants were called with Genome Analysis Toolkit (GATK). The detected variants were annotated with Ensembl and variants with genomic frequency MAF ≥ 0.01 in ExAC, gnomAD, or 1000 Genomes Project were excluded. Considering that the patient was from a consanguineous family, homozygous and hemizygous variants were retained for further screening. Variants affecting protein sequence and missense variants that were predicted to be deleterious by at least half of the software (SIFT, PolyPhen-2 HDIV, PolyPhen-2 HVAR, and Mutation Taster) were further kept. We next screened and kept genes that are expressed in human testis and function during spermatogenesis.

### PCR and Sanger sequencing

gDNA was extracted from human peripheral blood or hiPSCs and used to amplify *PIWIL2* fragment with TAKARA Ex Taq (TAKARA, Japan) on a SimpliAmp Thermal Cycler (Thermo Fisher Scientific, USA). The specific primers are listed in Additional file 4: Table S1. PCR products were sequenced on a 3730XL DNA Analyzer with forward primers, and the results were viewed on the Chromas.

### Histological analysis and immunohistochemistry

Testicular samples derived from testicular biopsy were fixed with 4% paraformaldehyde and embedded with paraffin. For histological analysis, testicular sections were prepared and stained with hematoxylin and eosin. For the detection of PIWIL2 protein, the testicular sections were prepared and blocked with 5% bovine serum albumin (BSA), followed by incubation with PIWIL2 or isotype (all from Abcam, UK) primary antibody (Additional file 4: Table S2) at 4 °C overnight. After incubation with a secondary antibody (Additional file 4: Table S2) at room temperature for 1 h, the sections were visualized with the use of the DAB Horseradish Peroxidase Color Development Kit (Beyotime, China). Nuclei were stained with hematoxylin. The sections were then observed under an Olympus microscope.

### Cell culture

The hiPSC line of the patient was generated from fibroblasts derived from a skin specimen of the testicular biopsy incision. The normal hiPSC line was generated from fibroblasts derived from the foreskin of a fertile volunteer. These two hiPSC lines have been described elsewhere [35, 36]. The homozygous c.731_732delAT variant in *PIWIL2* in the hiPSCs of the patient was confirmed using Sanger sequencing, and the normal hiPSCs were of wild-type at the same location [26]. The hiPSCs were seeded on Matrigel (Corning, USA)-covered plates and maintained in mTeSR 1 medium (STEMCELL Technologies, Canada) at 37 °C and 5% CO_2_, and the medium was changed every day. HiPSCs were passaged every 3–4 days at a ratio of 1:3 using 0.5 mM EDTA.

### Knockout of *PIWIL2* in normal hiPSCs

Single guide RNA (AGACCTCCGTTGGTTGGAGTAGG) plasmids (pX330) targeting exon 4 of *PIWIL2* were constructed. Plasmids (1 μg) were transfected into hiPSCs (2 × 10^5^/ per well of the 6-well plate) by 3 μL Lipofectamine Stem Transfection Reagent (Thermo Fisher Scientific, USA). Cells were allowed to grow into colonies from single cells and the colonies were manually picked for Sanger sequencing to recognize the homozygous and heterozygous mutant colonies. *PIWIL2*^−/−^ and PIWIL2^+/^^−^ colonies were retained for further studies.

### Western blot

Proteins from the testicular tissue and cells were extracted using RIPA buffer containing PMSF. After centrifuging at 12,000 ×*g* for 15 min at 4 °C, the supernatant containing proteins was mixed with loading buffer and denatured. Proteins were separated on 10% SDS-PAGE gel and transferred to PVDF membranes (Millipore, USA). The membranes were blocked with 5% BSA and incubated with PIWIL2 (Abcam, UK) and β-Actin (Proteintech, China) primary antibodies (Additional file 4: Table S2) at 4 °C overnight. The membranes were then incubated with secondary antibodies (Additional file 4: Table S2) at room temperature for 1 h and visualized using a BeyoECL Plus kit (Beyotime, China) on a ChemiDoc XRS + System (Bio-Rad, USA).

### Cell counting kit-8 assay

The cell proliferation rate was measured using the Cell Counting Kit-8 (Beyotime, China) in accordance with the manufacturer’s protocol.

### In vitro differentiation of hiPSCs into germ cell-like cells

In vitro differentiation of hiPSCs into PGCLCs and SSCLCs was performed as previously described [25, 26].

### Flow cytometry

PGCLCs were dissociated and stained with ITGA6 (BD, USA) and EpCAM (BD, USA) primary antibodies (Additional file 4: Table S2) [25]. SSCLCs were dissociated with Accutase (Thermo Fisher Scientific, USA) and stained with PLZF (Invitrogen, USA) primary antibody (Additional file 4: Table S2) after fixing, permeabilizing, and blocking processes [26]. The percentages of PGCLCs and SSCLCs were determined by a flow cytometer (Beckman Coulter, USA).

### RT-qPCR

Total RNA was extracted from cells using TRIzol (Thermo Fisher Scientific, USA) and then reverse-transcribed into cDNA using HiScript III RT SuperMix (Vazyme, China) on a SimpliAmp Thermal Cycler (Thermo Fisher Scientific, USA). RT-qPCR was conducted with ChamQ Universal SYBR qPCR Master Mix (Vazyme, China) on a StepOne Real-Time PCR system (Thermo Fisher Scientific, USA). Primers used in this study are listed in Additional file 4: Table S1. *β-Actin* was used as the internal control. Gene expression was assessed using the 2^−ΔΔCT^ method.

### Immunofluorescence

Cells were fixed with 4% paraformaldehyde for 15 min at room temperature. After washing with PBS, cells were blocked and permeabilized with 5% BSA containing 0.3% Triton X-100 for 45 min at room temperature. Cells were then incubated with PLZF (Santa Cruz, USA) and GPR125 (GeneTex, USA) primary antibodies (Additional file 4: Table S2) at 4℃ overnight, followed by incubation with secondary antibodies (Additional file 4: Table S2) in room temperature for 1 h. Nuclei were stained with DAPI. Cells were observed under a fluorescence microscope (Olympus, Japan).

### RNA-seq and data analysis

Total RNA was extracted from biological duplicates of cells from each group and used to construct RNA-seq libraries using a TruSeq Stranded mRNA Kit (Illumina, USA). Libraries were sequenced using the Illumina NovaSeq 6000 platform. After removing the adaptor sequence and low-quality reads, clean reads were mapped to the human genome GRCh38/hg38 using HISAT2. The aligned reads of genes were counted and normalized to evaluate gene expression as normalized counts per million by StringTie. Significantly differentially expressed genes were those with false discovery rate (FDR) < 0.05 and |log_2_(fold change)|> 1 assessed by edgeR. Pathway analyses were performed using KOBAS 3.0 (http://bioinfo.org/kobas) [37].

### Statistical analysis

Data are presented as mean ± SD. Statistical analyses were conducted with One-way ANOVA using GraphPad Prism 9, and figures were also created with this software. *P* < 0.05 was considered statistically significant.

## Results

### Clinical characterization of the man with complete SCOS

The clinical parameters of the patient (Fig. 1A, IV1) are summarized in Table 1. He had a normal karyotype (46, XY) and no microdeletions on the Y chromosome. The testicular volume on both sides was reduced, but serum hormone levels (total /free testosterone, follicle-stimulating hormone, and luteinizing hormone) were normal. Semen analyses indicated that no sperm was present. Additionally, increased seminal elastase level implied genital tract infection. Testicular histology showed that none of the observed seminiferous tubules contained spermatogenic cells, representing complete SCOS (Fig. 1D). The absence of seminal *DDX4* (a germ cell-specific gene) mRNA can serve to discriminate complete SCOS more accurately [5]. The seminal *DDX4* mRNA test result of the patient was negative, which confirmed the testicular histology of complete SCOS.

### Identification of potentially pathogenic variants from WES data

We performed a WES analysis of the patient to screen potentially pathogenic variants responsible for infertility. Considering the consanguineous background and the fact that the patient was the only infertile case in his family, we retained likely causative variants by filtering the WES data according to (i) genomic frequency MAF < 0.01, (ii) the recessive inheritance mode, and (iii) functional impact on protein sequence and predicted to be deleterious. Finally, eight potential disease-causing variants in eight genes (Additional file 4: Table S3) were identified for the manual screening of their expression in testis and roles in spermatogenesis. A frameshift deletion variant p.His244ArgfsTer31 (c.731_732delAT) mapped to the *PIWIL2* gene caught our attention because *PIWIL2* is the only gene that is expressed in testis and is associated with male infertility in mice among these eight genes [32, 33]. Spermatogenesis of *Mili*^−/−^ mice was blocked at the early prophase of the first meiosis, resulting in an incomplete SCO phenotype where most seminiferous tubules were depleted of spermatogonia [32, 33]. A homozygous missense variant (c.745C > T) and a homozygous non-frameshift deletion variant (c.727_729del) in *PIWIL2* in two NOA patients have been reported by Alhathal et al. [31], and Li et al. identified a homozygous missense variant (g.22145115C > T) in *PIWIL2* in an NOA patient [38], but the effects of these three variants on protein function were not as serious as the variant identified in our study. Therefore, the homozygous frameshift deletion variant in *PIWIL2* was suspected to be the causative variant of infertility in this patient. Sanger sequencing confirmed c.731_732delAT in *PIWIL2* in the patient, and his father and brother carried the same variant in heterozygosis (Fig. 1B), implying that his mother also carried the same heterozygous variant in *PIWIL2* and the inheritance mode of this variant was indeed autosomal recessive.

*PIWIL2* is located on human chromosome 8 and encodes a protein belonging to the PIWI family. The PIWIL2 protein consists of 973 amino acids (aa) and contains three domains, including Argonaute linker 1 domain (339.386 aa), PAZ domain (387.504 aa), and PIWI domain (512.956 aa). The conserved functions of the PAZ domain and PIWI domain are RNA binding and RNA silencing [39, 40], respectively. In this study, the identified variant in *PIWIL2* was a two-nucleotide deletion in exon 6, which introduced a premature stop codon and was predicted to result in a truncated protein of 273 aa that lacked all functional domains (Fig. 1C). We used a PIWIL2 antibody whose immunogen is in the middle part of the PIWIL2 protein (410.460 aa) to label PIWIL2 protein expressed in patient’s testes and human testes with normal spermatogenesis. No positive signal was observed in seminiferous tubules of the patient’s testicular tissues, but PIWIL2 was shown to be expressed in spermatogonium, spermatocytes, and round spermatids in the testes with normal spermatogenesis (Fig. 1E). Hence, the homozygous frameshift deletion variant in *PIWIL2* resulted in a truncated PIWIL2 protein that could not exert effects on human germ cells.

### The LoF variant in *PIWIL2* did not affect PGCLC specification

**Fig. 2:**
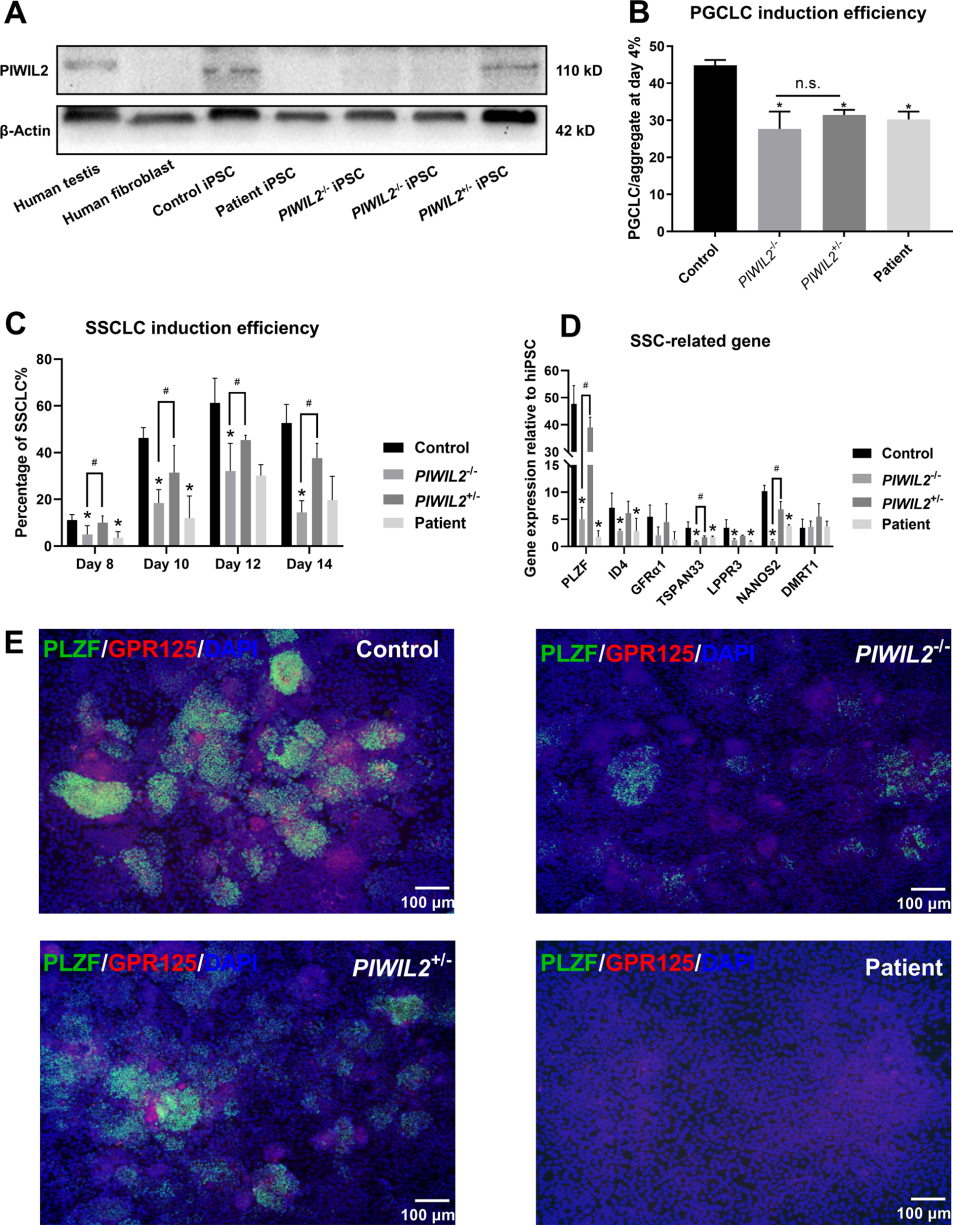
Differentiation of hiPSC lines into germ cell-like cells. **A** Western blot detected the expression of PIWIL2 using an antibody whose antigen was the middle part of PIWIL2 in human testis with normal spermatogenesis, human fibroblasts, and different hiPSC lines. Very faint band appeared in *PIWIL2*^−/−^ hiPSCs might because the antibody bound to nonspecific proteins. **B** PGCLCs were stained with ITGA6 and EpCAM and the percentage of PGCLCs reflecting the PGCLC induction efficiency was determined at 4 days of differentiation. n = 3, n.s., not significant. **C** SSCLCs were stained with PLZF and the percentage of SSCLCs reflecting the SSCLC induction efficiency was determined at 8, 10, 12, and 14 days of differentiation. n = 3, * versus Control, # versus *PIWIL2*^+/^^−^, *p* < 0.05. **D** Detection of SSC-related gens in the control, *PIWIL2*^−/−^, *PIWIL2*^+/^^−^ and patient groups by RT-qPCR at 12 days of differentiation. n = 3, * versus Control, # versus *PIWIL2*^+/^^−^, *p* < 0.05. **E** Immunofluorescence staining of PLZF (green) and GPR125 (red) in the control, *PIWIL2*^−/−^, *PIWIL2*^+/^^−^ and patient groups at 12 days of differentiation. The nuclei were stained with DAPI (blue). PLZF^+^/GPR125^+^ cells represented SSCLCs; they had relatively small nuclei and grew in clusters. There were less PLZF^+^/GPR125^+^ cells in the patient and *PIWIL2*^−/−^ groups, but cells with larger nuclei represent different differentiated cells

To validate the effects of the LoF variant in *PIWIL2* on human germ cells and explore the role of PIWIL2 in human germ cell development, we employed in vitro differentiation of hiPSCs into germ cell-like cell models. This approach circumvents the difficulties and ethical issues in obtaining human primary germ cells for functional experiments. The hiPSC line of the patient shares the same genetic background as its donor cell; it retains the homozygous c.731_732delAT variant in *PIWIL2* as in the patient with SCOS [36]. We also used CRISPR/Cas9 to introduce a homozygous frameshift deletion in *PIWIL2* (*PIWIL2*^−/−^) in the normal hiPSC line (control), leading to a truncated PIWIL2 protein (165 aa) nearly equivalent to the truncated PIWIL2 protein expressed in the testes of the patient (Additional file 1: Fig. S1A and B). The *PIWIL2*^+/−^ hiPSC line was also retained to simulate the variant carried by the patient’s father and brother (Additional file 1: Fig. S1B). The expression of PIWIL2 was confirmed in hiPSC lines using western blot (Fig. 2A). The expression of pluripotent genes and cell proliferation in *PIWIL2*^−/−^ and *PIWIL2*^+/^^−^ hiPSC lines were comparable to that in normal hiPSC line (Additional file 1: Fig. S1C).

PIWIL2 is first expressed in late PGCs [34]. We explored whether the LoF variant in *PIWIL2* affected human PGCs using a known in vitro differentiation model of differentiating hiPSCs into PGCLCs [35]. At 4 days of differentiation when PGCLCs were abundant, the percentage of PGCLCs was decreased in the *PIWIL2*^−/−^, *PIWIL2*^+/^^−^, and patient groups compared with the control group (*p* < 0.05), but there was no significant difference between the *PIWIL2*^−/−^ and *PIWIL2*^+/^^−^ groups (Fig. 2B, Additional file 1: Fig. S1D). Thus, the LoF variant in *PIWIL2* and deletion of PIWIL2 did not affect the specification of human PGCs.

### The LoF variant in *PIWIL2* affected SSCLC formation and maintenance

We further differentiated hiPSCs into SSCLCs to investigate whether the LoF variant in *PIWIL2* could cause abnormalities in human SSCs. The SSCLC induction efficiency was reduced in the *PIWIL2*^−/−^ and patient groups at 8, 10, 12, and 14 days of differentiation when compared with the control group, and the induction efficiency in the *PIWIL2*^−/−^ group was also lower than that in the *PIWIL2*^+/^^−^ group (*p* < 0.05) (Fig. 2C, Additional file 2: Fig. S2). RT-qPCR was performed to determine the expression of SSC-related genes after 12 days of differentiation. Similar to the trends of SSCLC induction efficiency, the expression of SSC-related genes, including *PLZF*, *ID4*, *GFRα1*, *TSPAN33*, *LPPR3*, and *NANOS2* was significantly lower in the patient and *PIWIL2*^−/−^ groups compared with the control group, and the gene expression in the *PIWIL2*^−/−^ group was also significantly lower than that in the *PIWIL2*^+/^^−^ group (Fig. 2D). Immunofluorescence results showed that the staining of co-expressed PLZF and GPR125, which represented SSCLCs, was weak and less intensive in the patient and *PIWIL2*^−/−^ groups than in the control and *PIWIL2*^+/^^−^ groups after 12 days of differentiation (Fig. 2E). Moreover, from 12 to 14 days of differentiation, the induction efficiency in the control and *PIWIL2*^+/^^−^ groups remained at a stable level, whereas the induction efficiency in the patient and *PIWIL2*^−/−^ groups declined (Fig. 2C). These results suggested that the LoF variant in *PIWIL2* and deletion of PIWIL2 affected the formation and maintenance of SSCLCs.

### Transcriptome analyses of hiPSCs and cells at 8 and 12 days of SSCLC induction

Next, we analyzed the transcriptome of hiPSCs (day 0 of differentiation) and cells at 8 and 12 days of SSCLC induction from the control and *PIWIL2*^−/−^ groups. In hiPSCs, only 136 differentially expressed genes (DEGs) were found between the control and *PIWIL2*^−/−^ groups (Additional file 3: Fig. S3A, Additional file 5: Table S4). At 8 days of differentiation, when SSCLCs emerged, 6125 DEGs were found in the control group (3475 upregulated and 2650 downregulated) and 4472 DEGs were found in the *PIWIL2*^−/−^ group (2791 upregulated and 1681 downregulated) compared with corresponding hiPSCs (Additional file 3: Fig. S3B). At 12 days of differentiation, more DEGs were identified. There were 4489 upregulated genes and 3213 downregulated genes in the control group, and 3831 upregulated genes and 2186 downregulated genes in the *PIWIL2*^−/−^ group, compared with the corresponding hiPSCs (Additional file 3: Fig. S3C). SSCLC induction process induced a global change in the transcriptome, but deletion of PIWIL2 seemed to affect gene expression profile.

**Fig. 3:**
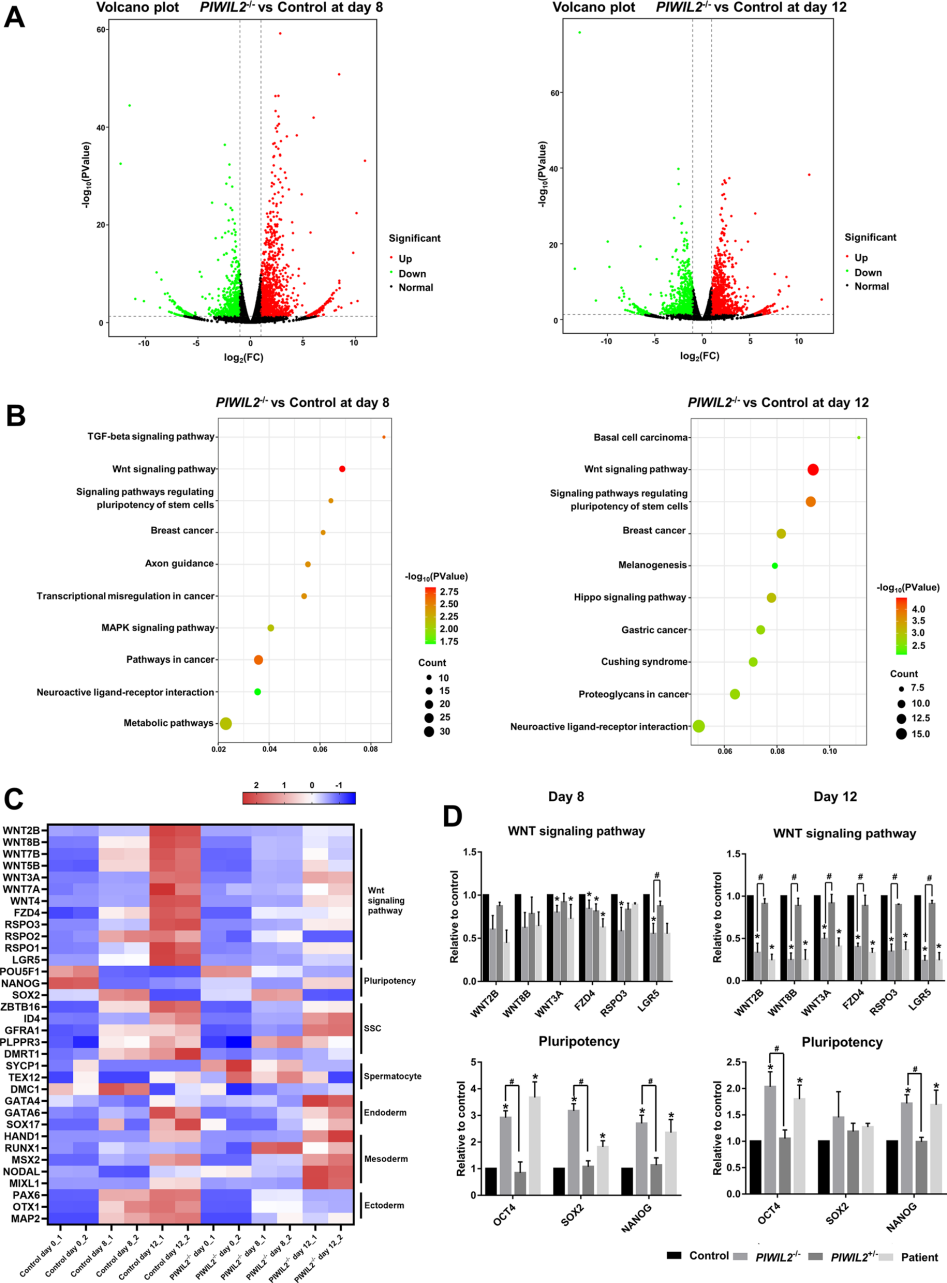
Transcriptome analysis of cells at 0, 8, and 12 days of SSCLC induction. **A** Volcano plots of comparison of transcriptome between the control and *PIWIL2*^−/−^ groups at 8 and 12 days of differentiation. Red dots represented upregulated genes, green dots represented downregulated genes, and black dots represented genes not significantly changed. **B** KEGG analysis on the downregulated genes at 8 and 12 days of differentiation. **C** The expression of genes in Wnt signaling pathway, pluripotency, SSC, spermatocyte, endoderm, mesoderm, and ectoderm in the control and *PIWIL2*^−/−^ groups at 0, 8, and 12 days of differentiation. **D** RT-qPCR confirmed the expression of genes in Wnt signaling pathway and pluripotency in the control and *PIWIL2*^−/−^ groups at 8 and 12 days of differentiation. n = 3, * versus Control, # versus *PIWIL2*^+/^^−^, *p* < 0.05

We compared the transcriptome of the control and *PIWIL2*^−/−^ groups at 8 and 12 days of SSCLC induction. At 8 days of differentiation, there were 1200 upregulated genes and 786 downregulated genes between these two groups (Fig. 3A). At 12 days of differentiation, there were 1114 upregulated genes, but the number of downregulated genes increased to 981 (Fig. 3A). KEGG analyses revealed that DEGs were involved in the TGF-beta signaling pathway, MAPK signaling pathway, Hippo signaling pathway, and Wnt signaling pathway (Fig. 3B, Additional file 3: Fig. S3D). We noticed that the downregulated genes in the control and *PIWIL2*^−/−^ groups were both enriched in the “Wnt signaling pathway” and “Signaling pathways regulating pluripotency of stem cells” at 8 and 12 days of differentiation (Fig. 3B). The Wnt signaling pathway genes were downregulated but pluripotent genes were upregulated in the *PIWIL2*^−/−^ group compared with the control group (Fig. 3C). Additionally, SSC-related genes were downregulated in the *PIWIL2*^−/−^ group, but the expression of genes related to endoderm and mesoderm was not obviously altered (Fig. 3C). Transcriptome analysis indicated that deletion of PIWIL2 failed to induce the expression of the Wnt signaling pathway genes and SSC genes and repress pluripotent genes during differentiation, but the development of germ layers was not affected. We confirmed the expression of genes in the Wnt signaling pathway and pluripotency in the control and *PIWIL2*^−/−^ groups at 8 and 12 days of differentiation by RT-qPCR, and the results showed the same trends as the transcriptome analysis (Fig. 3D). Decreased expression of genes in the Wnt signaling pathway and increased expression of pluripotent genes were also confirmed in the patient group, and the trends of gene expression in the *PIWIL2*^+/^^−^ group were similar to those in the control group (Fig. 3D).

### Inactivated Wnt signaling pathway might affect SSCLC formation and maintenance

**Fig. 4:**
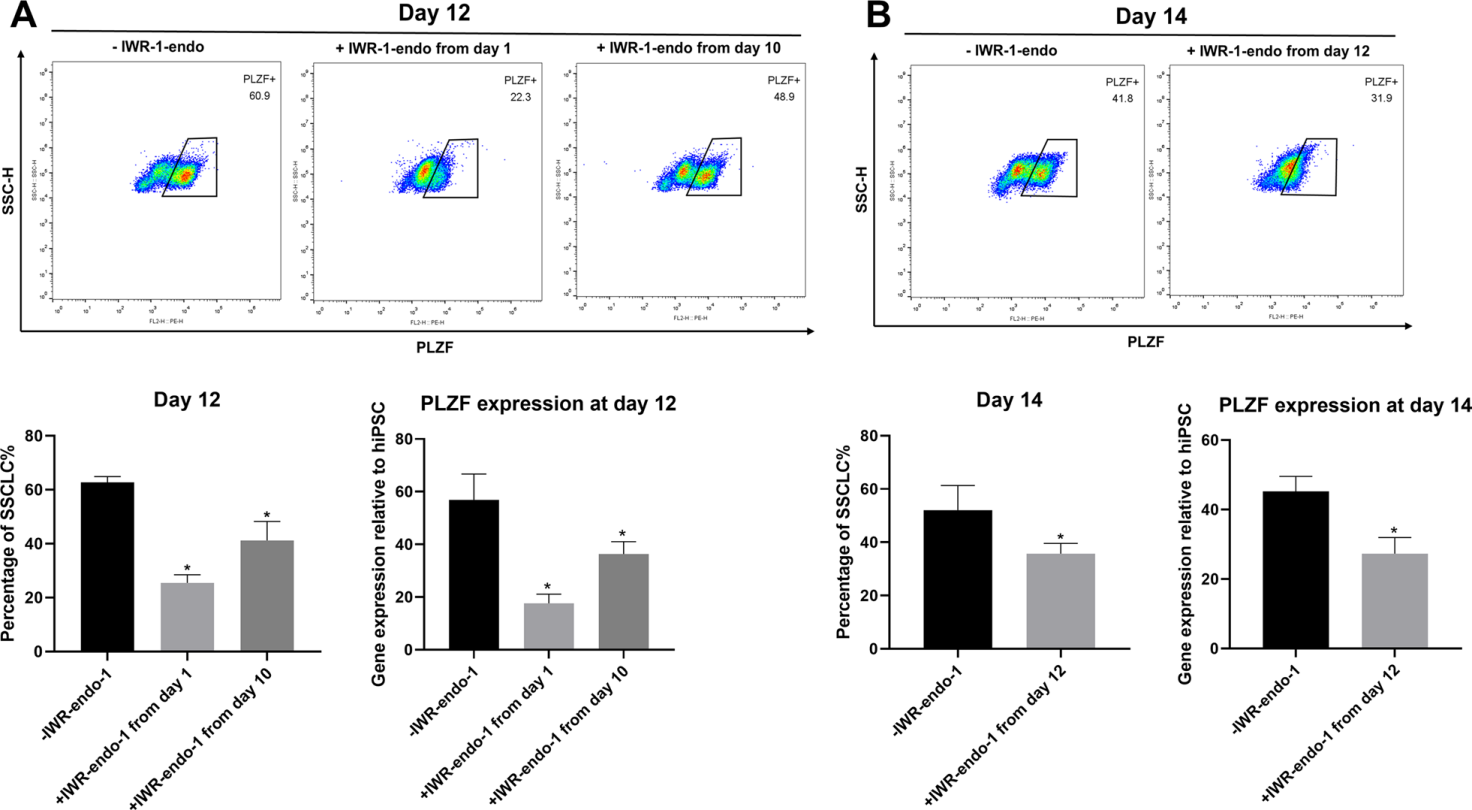
Inhibition of Wnt signaling pathway impaired formation and maintenance of SSCLCs. **A** Wnt signaling pathway inhibitor IWR-endo-1 was added into the SSCLC induction medium from 1 day or 10 days of differentiation, and the percentage of SSCLCs was determined at 12 days of differentiation. The expression of *PLZF* was also determined by RT-qPCR at 12 days of differentiation. n = 3, * versus −IWR-endo-1, *p* < 0.05. **B** Wnt signaling pathway inhibitor IWR-endo-1 was added into the SSCLC induction medium from 12 days of differentiation, and the percentage of SSCLCs was determined at 14 days of differentiation. The expression of *PLZF* was also determined by RT-qPCR at 14 days of differentiation. n = 3, * versus IWR-endo-1, *p* < 0.05

We suspected that the impaired formation and maintenance of SSCLCs might be associated with the inactivated Wnt signaling pathway. We added IWR-endo-1 (a Wnt signaling pathway inhibitor) to the SSCLC induction medium of the control group from days 1, 10, and 12 of differentiation and detected the SSCLC induction efficiency and the expression of *PLZF* on 12 or 14 days of differentiation. When IWR-endo-1 was added to the medium from day 1, the SSCLC induction efficiency significantly decreased compared with normal differentiation on day 12 (*p* < 0.05) (Fig. 4A). Two days of treatment with IWR-endo-1 from day 10 to day 12 also reduced the SSCLC induction efficiency detected on day 12 (*p* < 0.05) (Fig. 4A). Moreover, treatment with IWR-endo-1 from day 12 reduced the percentage of SSCLCs detected on day 14 (*p* < 0.05) (Fig. 4B). These results indicated that inhibition of the Wnt signaling pathway impaired the formation and maintenance of SSCLCs. Deletion of PIWIL2 might inactivate the Wnt signaling pathway, thereby abolishing the formation and maintenance of SSCLCs.

## Discussion

In this study, we performed WES on a man with complete SCOS and identified the LoF variant p.His244ArgfsTer31 (c.731_732delAT) in *PIWIL2*. We further revealed that the LoF variant in *PIWIL2* could impair the formation and maintenance of human SSCLCs, which was associated with the inactivation of the Wnt signaling pathway. Clinical and functional evidence suggested *PIWIL2* as a causative gene of SCOS. Our study reinforced the understanding that the etiology of NOA involves multiple genes, and we provided a new gene for SCOS.

To date, the association between *PIWIL2* and infertility in humans has not yet been fully explained. Alhathal et al. found a homozygous missense variant (c.745C > T) and a homozygous non-frameshift deletion variant (c.727_729del) in *PIWIL2* in two independent patients with NOA [31], and Li et al. identified a homozygous missense variant (g.22145115C > T) in an NOA patient [38], but these three variants have not been validated by functional experiments or in a larger group of infertile men. Other studies have shown that SNPs in *PIWIL2* (rs4871990 and rs13259097 in the promoter) were not associated with the risk of azoospermia and oligozoospermia, but SNPs in the other two members of PIWI family, *PIWIL4* (rs508485) and *PIWIL3* (rs11703684), were significantly associated with the risk of oligozoospermia [41–43]. Our study reported a homozygous frameshift deletion variant in *PIWIL2* in a man with complete SCOS, and this variant greatly affected the function of PIWIL2. The patient’s father and brother were heterozygous carriers, suggesting that the heterozygous frameshift variant in *PIWIL2* may not affect male infertility.

The current knowledge of PIWIL2 in spermatogenesis originates mainly from mouse models. PIWIL2 (MILI) is the earliest PIWI protein expressed in male germ cells from the late PGC stage to the round spermatid stage in mice [44]. MILI mainly performs the homotypic ping-pong cycle to silence transposons in a piRNA-dependent manner and produce piRNAs that associate with MIWI2 [44]. SSCs of *Mili*^−/−^ mice could not complete self-renewal and differentiation; hence, spermatogenesis was blocked at the early prophase of the first meiosis, and most seminiferous tubules were depleted of spermatogonia by 180 days postpartum, resulting in an incomplete SCO phenotype [32, 33]. The SCO phenotype of *Mili*^−/−^ mice was similar to that of the patient included in our study, suggesting that the LoF variant in *PIWIL2* affected male infertility and caused SCOS phenotype. However, the testicular phenotype of the patient was complete SCOS, which was different from the incomplete SCO in *the Mili*^−/−^ mice. This difference implies that the impairment in human SSCs of the patient may have occurred earlier than that in mice.

Meiotic arrest in mice caused by knockout of *Mili* was suggested to be associated with desuppression of transposons through piRNA pathway [45]. In humans, PIWIL2 is expressed in the cytoplasm of PDPN^+^/DDX4^−^ germ cells (late PGC stage) and in cytoplasm and nuclei of some PDPN^−^/DDX4^+^ germ cells (fetal spermatogonia stage), spermatogonium and spermatocytes, and strongly accumulates in nuclei of spermatids [34]. In mice, MILI is expressed in the cytoplasm of male germ cells [34]. Moreover, PIWIL4, but not PIWIL2, is present in intermitochondrial cement of human PDPN^−^/DDX4^+^ germ cells [34]. In contrast, in mice, MILI exists in intermitochondrial cement, whereas MIWI2 is in piP-bodies [34]. The different expression patterns and locations of PIWIL2 between humans and mice suggested that the exact role of PIWIL2 in germ cell development and spermatogenesis might be different between these two species.

To determine whether the LoF variant in *PIWIL2* is indeed the cause of SCOS, we used in vitro differentiation models to generate human germ cell-like cells from hiPSCs. Very small embryonic-like stem cells (VSELs) which express pluripotent and PGC-specific markers have been found in SCOS testis [46, 47], but these cells account for only a very small fraction of the total number of testicular cells (0.03% of total cells of normal mouse testis [48]) and are difficult to isolate [46, 47]. Although VSELs have the potential to be differentiated into germ cells [47, 48], hiPSCs are more easily to be expanded in vitro. The testicular biopsy tissue of the patient was used for histological analysis because the initial purpose of the patient was clinical diagnosis and artificial reproduction technology consulting and therefore could not be used to isolate VSELs. In our study, we differentiated hiPSCs into germ cell-like cells in vitro. In vitro models circumstance the problem that human germ cells are difficult to obtain, purify, and culture, and target deletion or over-expression of genes can be easily performed in this system [49]. In vitro models could be a new approach to directly study human germ cells and identify unique mechanisms in human reproduction [49]. The combination of this approach and animal models provided more evidence for the assessment of gene–disease associations [50]. Our findings demonstrated that the LoF variant in *PIWIL2* and the homozygous deletion of *PIWIL2* did not affect the specification of PGCLCs but impaired the formation and maintenance of SSCLCs. These findings provided direct evidence that the LoF variant in *PIWIL2* affected the formation and maintenance of human SSCs and thus caused SCOS. Interestingly, deletion of MILI in mice did not affect the formation of SSC population but hindered the SSC self-renewal and differentiation into pachytene spermatocytes [32, 33], which was different from the results of our in vitro differentiation model. Our results supported the possibility that PIWIL2 might have different roles in human spermatogenesis and revealed its importance in the formation and maintenance of human SSCs.

The Wnt signaling pathway plays important roles in embryonic germ cells development and spermatogenesis in mice [51] and regulates the self-renewal and proliferation of mouse SSCs [52–54]. The Wnt signaling pathway activates EMOES, which in turn induces human PGCLC specification by upregulating SOX17 [55]. The role of the Wnt signaling pathway in the formation, maintenance, and differentiation of human SSCs has been largely unclear. Using an in vitro differentiation model, our study proposed that the Wnt signaling pathway was involved in the formation and maintenance of human SSCs, and deletion of PIWIL2 affected the expression of genes in the Wnt signaling pathway, which might in turn impair the formation and maintenance of human SSCs. Hence, the Wnt signaling pathway might be a target to further investigate male infertility and develop relevant therapies to reconstruct spermatogenesis.

## Conclusions

In summary, our study reported the genotype–phenotype correlation of the LoF variant in *PIWIL2* and provided direct evidence that the LoF variant in *PIWIL2* impaired the formation and maintenance of human SSCs using in vitro differentiation model. Our study provided new evidence that *PIWIL2* is a male infertility gene in humans. Our results highlighted the application of in vitro differentiation models to functional experiments; using these models, we proposed the role of PIWIL2 in the formation and maintenance of human SSCs, which was different from that of mouse MILI. We showed that the Wnt signaling pathway was dysregulated during SSCLC induction from the patient and *PIWIL2*^−/−^ hiPSCs. Further research on the mechanism linking PIWIL2 and Wnt signaling is warranted. Furthermore, independent studies are needed to validate the correlation between *PIWIL2* and SCOS, before *PIWIL2* could be considered for clinical genetic tests and counseling. Whether *PIWIL2* variants affect VSELs in testis and therapeutic methods based on such cells are also worth exploring, since VSELs are good candidates for regenerative and reproductive medicine [46, 47, 56].

## Supplementary Information

**Additional file 1: Fig. S1.** Deletion of PIWIL2 in normal hiPSCs and differentiation of hiPSCs into PGCLCs. **A** sgRNA targeted exon 4 of *PIWIL2* which was supposed to introduce a premature stop codon and result in a truncated PIWIL2 protein. **B** Sanger sequencing confirmed the homozygous knockout of *PIWIL2* in *PIWIL2*^−/−^ hiPSCs and heterozygous knockout of *PIWIL2* in *PIWIL2*^+/^^−^ hiPSCs. **C** The expression of pluripotent genes (*OCT4*, *SOX2*, and *NANOG*) and transposon (LINE-1) in different hiPSC lines detected by RT-qPCR. Cell proliferation of different hiPSC lines detected by CCK8. **D** PGCLCs were stained with ITGA6 and EpCAM and the percentage of PGCLCs reflecting the PGCLC induction efficiency was determined at 4 days of differentiation.

**Additional file 2: Fig. S2.** The SSCLC induction efficiency of different hiPSC lines determined by flow cytometry. SSCLCs were stained with PLZF and the percentage of SSCLCs reflecting the SSCLC induction efficiency was determined at 8, 10, 12, and 14 days of differentiation.

**Additional file 3: Fig. S3.** Differentially expressed genes between control and *PIWIL2*^−/−^ groups at day 0, 8, and 12 days of SSCLC induction. **A** Differentially expressed genes (DEGs) between control and *PIWIL2*^−/−^ hiPSCs (day 0). **B** At 8 days of differentiation, compared with corresponding hiPSCs, 6125 and 4472 DEGs were found in the control and *PIWIL2*^−/−^ groups. **C** At 12 days of differentiation, 7702 and 6017 DEGs existed in the control and *PIWIL2*^−/−^ groups, compared with the corresponding hiPSCs. Red dots represented upregulated genes, green dots represented downregulated genes and black dots represented genes not significantly changed. **D** DEGs in Hippo signaling pathway, MAPK signaling pathway, and TGF-β signaling pathway in the control and *PIWIL2*^−/−^ groups at 0, 8, and 12 days of SSCLC induction.

**Additional file 4: Table S1.** Primers used in this study; **Table S2** Antibodies used in this study; **Table S3** Potential pathogenic variants detected in the man with complete SCOS.

**Additional file 5: Table S4.** DEGs between control and *PIWIL2*^-/-^ hiPSCs.

## Data Availability

The datasets supporting the conclusions of this article are included in this article.

## Abbreviations

DEG: Differentially expressed gene
FDR: False discovery rate
hiPSC: Human-induced pluripotent stem cell
LoF: Loss-of-function
MAF: Minor allele frequency
NOA: Non-obstructive azoospermia
PGCLC: Primordial germ cell-like cell
SCOS: Sertoli cell-only syndrome
SSCLC: Spermatogonial stem cell-like cells
VSEL: Very small embryonic-like stem cell
WES: Whole-exome sequencing
