# Supplementary figures and images for: A homozygous PIWIL2 frameshift variant affects the formation and maintenance of human-induced pluripotent stem cell-derived spermatogonial stem cells and causes Sertoli cell-only syndrome
Source: Stem Cell Res Ther. 2022 Sep 24;13:480. doi: 10.1186/s13287-022-03175-6 (PMC9509617; doi:10.1186/s13287-022-03175-6)

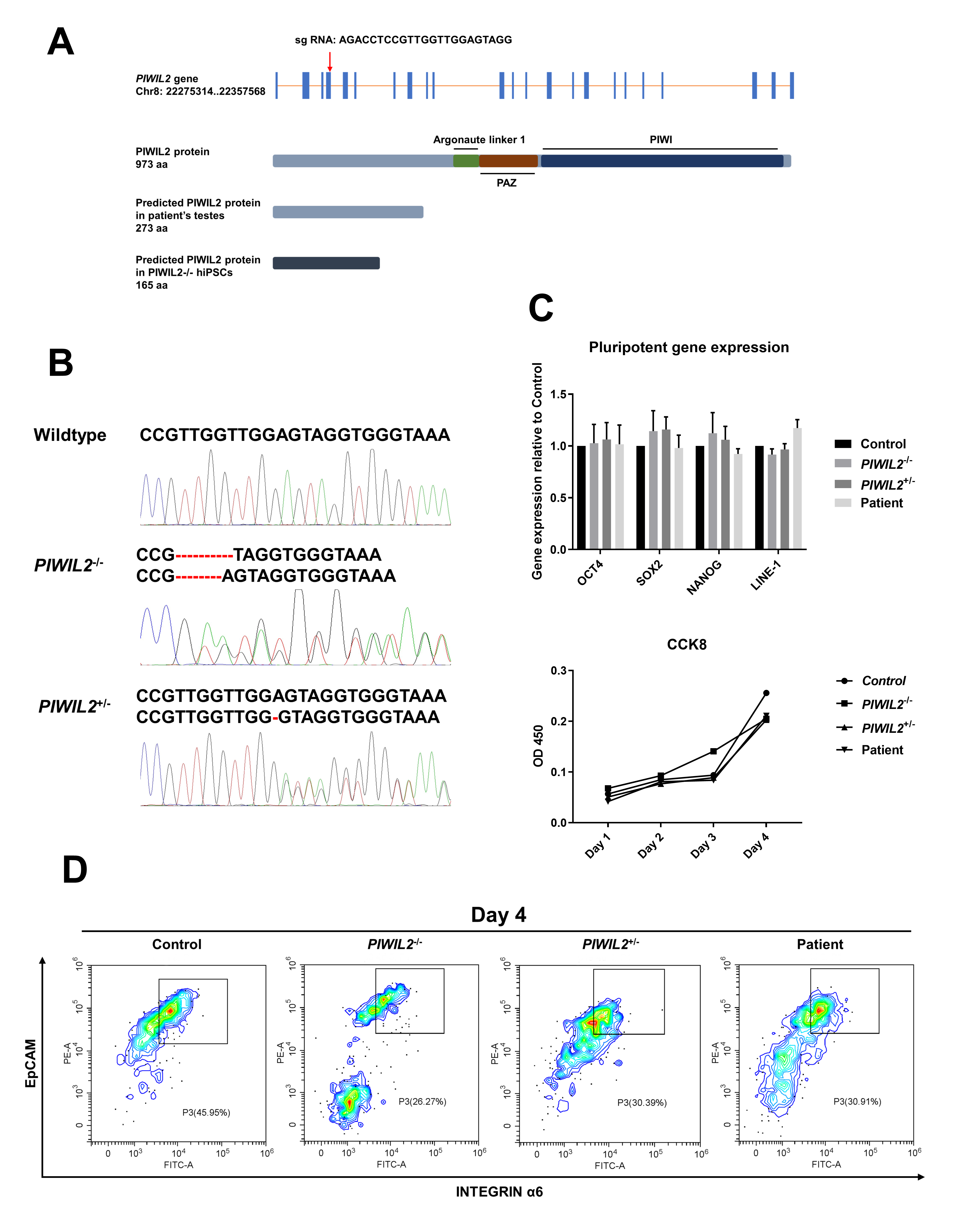

Supplement: Supplementary file 1 — Additional file 1: Fig. S1. Deletion of PIWIL2 in normal hiPSCs and differentiation of hiPSCs into PGCLCs. A sgRNA targeted exon 4 of PIWIL2 which was supposed to introduce a premature stop codon and result in a truncated PIWIL2 protein. B Sanger sequencing confirmed the homozygous knockout of PIWIL2 in PIWIL2−/− hiPSCs and heterozygous knockout of PIWIL2 in PIWIL2+/− hiPSCs. C The expression of pluripotent genes (OCT4, SOX2, and NANOG) and transposon (LINE-1) in different hiPSC lines detected by RT-qPCR. Cell proliferation of different hiPSC lines detected by CCK8. D PGCLCs were stained with ITGA6 and EpCAM and the percentage of PGCLCs reflecting the PGCLC induction efficiency was determined at 4 days of differentiation. [file 13287_2022_3175_MOESM1_ESM.tif]

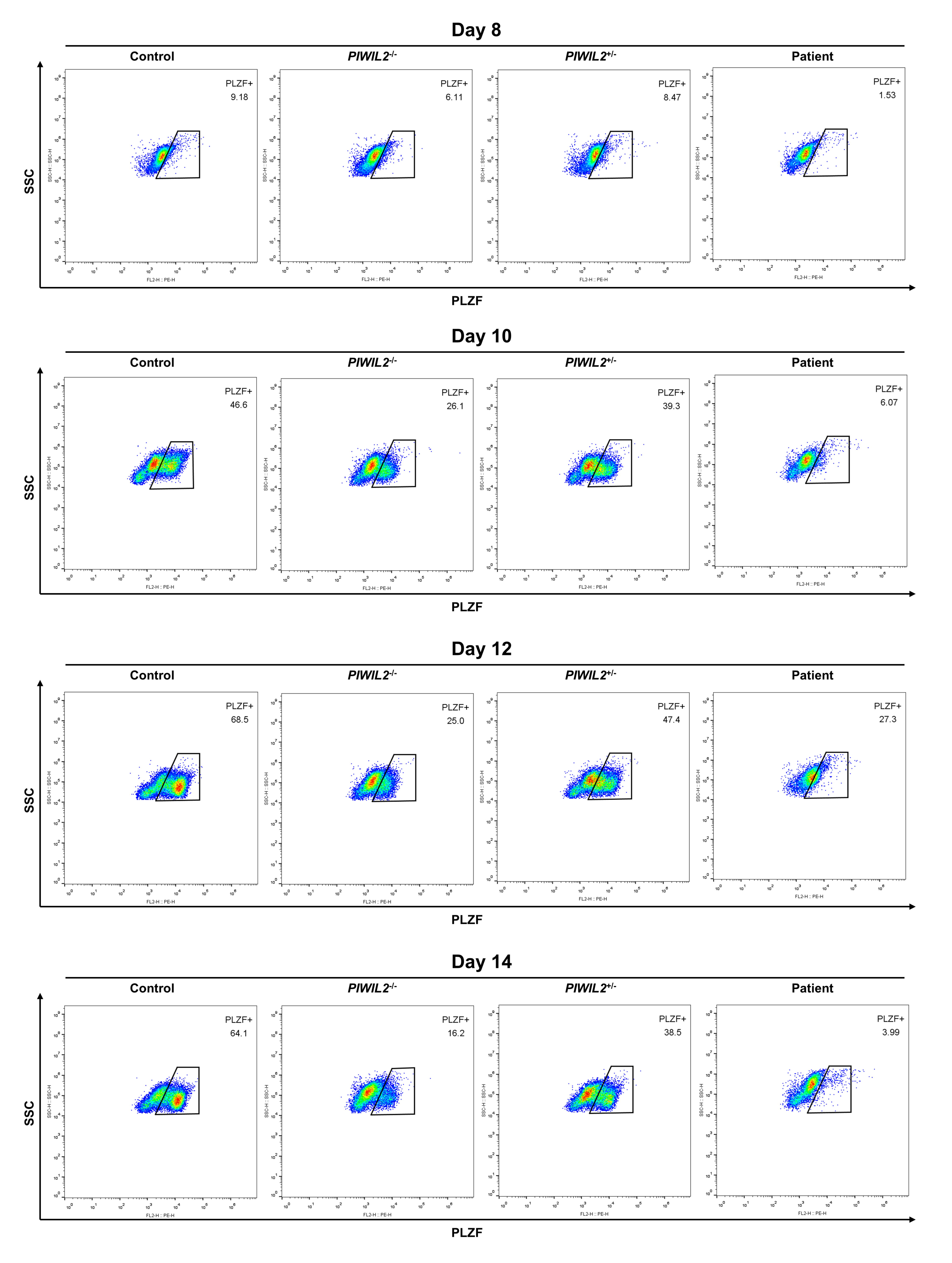

Supplement: Supplementary file 2 — Additional file 2: Fig. S2. The SSCLC induction efficiency of different hiPSC lines determined by flow cytometry. SSCLCs were stained with PLZF and the percentage of SSCLCs reflecting the SSCLC induction efficiency was determined at 8, 10, 12, and 14 days of differentiation. [file 13287_2022_3175_MOESM2_ESM.tif]

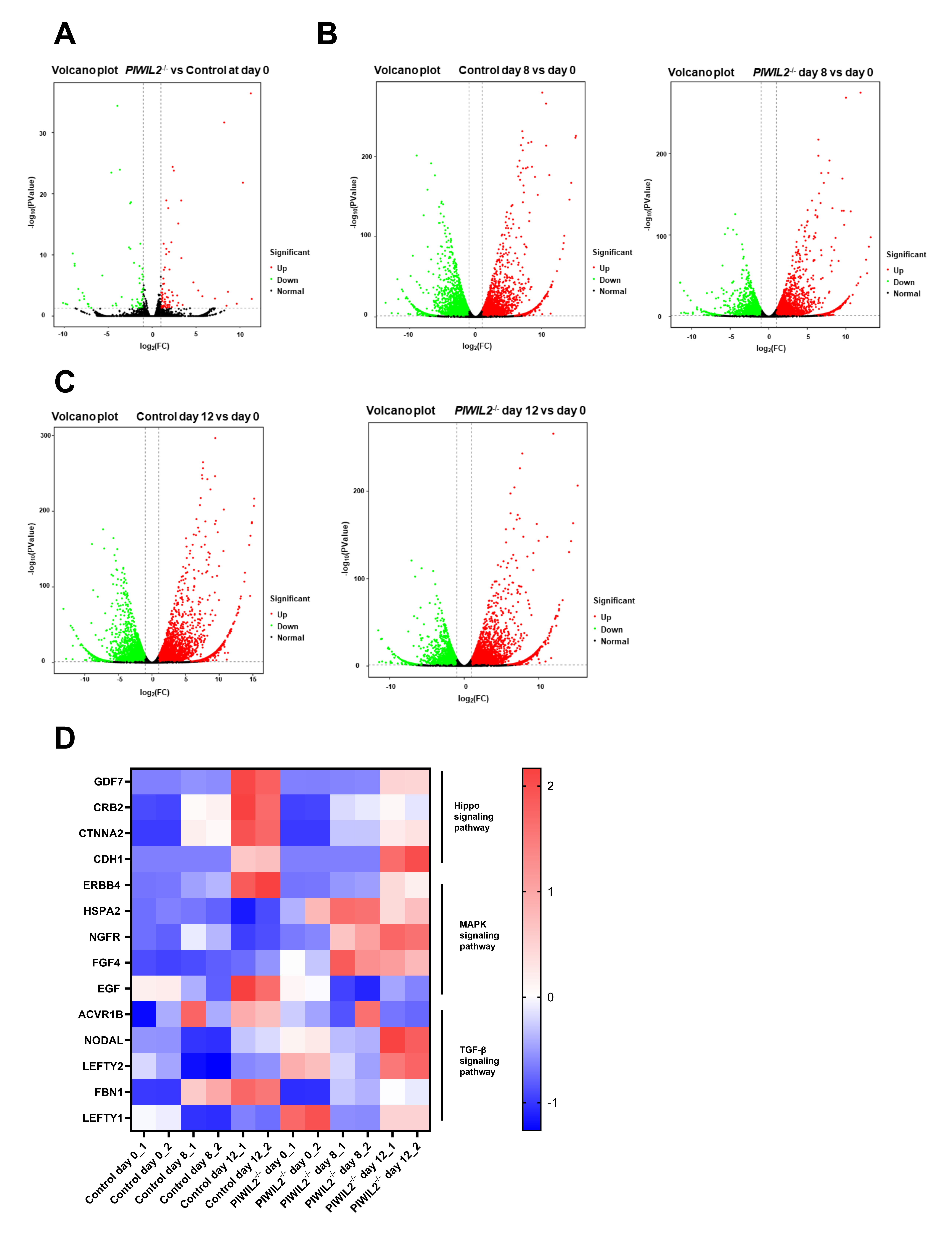

Supplement: Supplementary file 3 — Additional file 3: Fig. S3. Differentially expressed genes between control and PIWIL2−/− groups at day 0, 8, and 12 days of SSCLC induction. A Differentially expressed genes (DEGs) between control and PIWIL2−/− hiPSCs (day 0). B At 8 days of differentiation, compared with corresponding hiPSCs, 6125 and 4472 DEGs were found in the control and PIWIL2−/− groups. C At 12 days of differentiation, 7702 and 6017 DEGs existed in the control and PIWIL2−/− groups, compared with the corresponding hiPSCs. Red dots represented upregulated genes, green dots represented downregulated genes and black dots represented genes not significantly changed. D DEGs in Hippo signaling pathway, MAPK signaling pathway, and TGF-β signaling pathway in the control and PIWIL2−/− groups at 0, 8, and 12 days of SSCLC induction. [file 13287_2022_3175_MOESM3_ESM.tif]
