# Supplementary material for: A homozygous PIWIL2 frameshift variant affects the formation and maintenance of human-induced pluripotent stem cell-derived spermatogonial stem cells and causes Sertoli cell-only syndrome
Source: Stem Cell Res Ther. 2022 Sep 24;13:480. doi: 10.1186/s13287-022-03175-6 (PMC9509617; doi:10.1186/s13287-022-03175-6)
Supplement: Supplementary file 4 — Additional file 4: Table S1. Primers used in this study; Table S2 Antibodies used in this study; Table S3 Potential pathogenic variants detected in the man with complete SCOS. [file 13287_2022_3175_MOESM4_ESM.docx]

**Additional File Table 1 Primers used in this study**

| **Primers** | **Sequences** |
| --- | --- |
| *PLZF* | GAGATCCTCTTCCACCGCAAT/ CCGCATACAGCAGGTCATC |
| *ID4* | TCCCGCCCAACAAGAAAGTC/ CTGCAGGTCCAGGATGTAGTC |
| *GFRα1* | TGGAGCACATTCCCAAAGGG/ AGCATTCCGTAGCTGTGCTT |
| *NANOS2* | TCGCAAGTCGGGGTCAAAG/ CTGGTGTGAGGAGTAGACGTG |
| *TSPAN33* | AGCCCGCTGGTGAAATACCT/ TAGGGCTGCTTCTGCATGCTT |
| *LPPR3* | GCTATGACCGCACTCTCTCC/ ACAGTACAACATGCCCTCGG |
| *DMRT1* | CCAGCCGTCTCTGTTTCCTT/ CCCCAGAAGCAGAATCAGCA |
| *OCT4* | GACAGGGGGAGGGGAGGAGCTAGG/ CTTCCCTCCAACCAGTTGCCCCAAAC |
| *SOX2* | GGGAAATGGGAGGGGTGCAAAAGAGG/ TTGCGTGAGTGTGGATGGGATTGGTG |
| *NANOG* | ACCAGTCCCAAAGGCAAACA/ TCTGCTGGAGGCTGAGGTAT |
| *WNT3A* | TGCTGGACAAAGCTACCAGG/ CGAGACACCATCCCACCAAA |
| *WNT2B* | CCGAGAGTGTCAGCACCAAT/ CGCGAGTAATAGCGTGGACT |
| *WNT8B* | ACAGCTGGTCGGTGAACAAT/ CTGCCACACTGCTGGAGTAA |
| *RSPO3* | ATACATCGGCAGCCAAAACG/ CTTCCAACCCTTCTGGGCAA |
| *LGR5* | CATCAGCTATGTGCCCCCAA/ TGTGGAGCCCATCAAAGCAT |
| *β-Actin* | CTCCATCCTGGCCTCGCTGT/ GCTGTCACCTTCACCGTTCC |
| *PIWIL2* (for Sanger sequencing on the patient and his family members) | CAAGGATCAAAAGGAACACCTC/ TAATGCAAGTTTTAAAAGGCCC |
| *PIWIL2* (for Sanger sequencing on hiPSCs) | GTCGAGGCTTGTCTGCTAATCT/ GGGAGGCTTGTCCACTTCTC |

**Additional File Table 2 Antibodies used in this study**

| **Antibodies** | **Vendor** | **Dilution rate** |
| --- | --- | --- |
| Primary antibodies |  |  |
| PLZF mouse monoclonal antibody | Santa Cruz, sc-28319 | 1:150 |
| GPR125 rabbit polyclonal antibody | GeneTex, GTX51219 | 1:150 |
| PIWIL2 rabbit polyclonal antibody | Abcam, ab181340 | 1:150 |
| β-Actin mouse monoclonal antibody | Proteintech, 66009-1-Ig | 1:3000 |
| Rabbit IgG, polyclonal - Isotype Control | Abcam, ab37415 | 1:150 |
| PLZF mouse monoclonal antibody-PE | Invitrogen, 12-9320-82 | 1:200 |
| ITGA6 rat monoclonal antibody-FITC | BD, 555735 | 1:400 |
| EpCAM mouse monoclonal antibody-PE-CF594 | BD, 565399 | 1:400 |
| Secondary antibodies |  |  |
| CoraLite594 conjugate Goat anti-Rabbit IgG | Proteintech, SA00013-4 | 1:400 |
| CoraLite488 conjugate Goat anti-Mouse IgG | Proteintech, SA00013-1 | 1:400 |
| HRP-conjugated Affinipure Goat Anti-Rabbit IgG | Proteintech, SA00001-2 | 1:5000 |
| HRP-conjugated Affinipure Goat Anti-Mouse IgG | Proteintech, SA00001-1 | 1:5000 |

**Additional File Table 3 Potential pathogenic variants detected in the man with complete SCOS**

| **Gene** | **Chromosome** | **Variant** | **Reference SNP** | **ExAC_eas** | **1000 genome_eas** | **gnomAD_eas** | **SIFT** | **Polyphen2_HDIV** | **Polyphen2_HVAR** | **Mutation**  **Taster** |
| --- | --- | --- | --- | --- | --- | --- | --- | --- | --- | --- |
|  |  |  |  |  |  |  |  |  |  |  |
| *SLC19A3* | 2 | g.228560707G>A | rs778139709 | 0.0000 | - | 0.0008 | D | D | D | D |
| *ACKR3* | 2 | g.237489793G>A | rs200775693 | 0.0023 | 0.0000 | 0.0028 | D | D | D | D |
| *COL6A3* | 2 | g.238275918C>T | rs114322958 | 0.0057 | 0.0097 | 0.0063 | D | D | D | D |
| *PIWIL2* | 8 | g.22141773..22141774delAT | NA | - | - | - | - | - | - | - |
| *TSTA3* | 8 | g.144698374G>A | rs139998516 | 0.0015 | 0.0000 | 0.0014 | D | D | D | D |
| *PKP3* | 11 | g.399181C>T | rs140003591 | 0.0036 | 0.0049 | 0.0030 | D | D | D | D |
| *ZNF544* | 11 | g.58788583..58788630delGGCCAGAAGGATCTGAGAGAGTGTGGCCAGCTTGAGCCCTCAGGAGTC | NA | - | - | - | - | - | - | - |
| *FRMPD3* | X | g.106788994G>A | rs189007704 | 0.0049 | 0.0000 | 0.0045 | D | - | - | D |
